# Mechanisms of action of inhaled fibers, particles and nanoparticles in lung and cardiovascular diseases

**DOI:** 10.1186/1743-8977-4-4

**Published:** 2007-05-30

**Authors:** Brooke T Mossman, Paul J Borm, Vincent Castranova, Daniel L Costa, Kenneth Donaldson, Steven R Kleeberger

**Affiliations:** 1 Department of Pathology, University of Vermont, 89 Beaumont Avenue, HSRF 218, Burlington, VT 05405, USA; 2 University of Heerlen, CEL, Nieuw Eyckholt 300, Heerlen, 6400 AN, The Netherlands; 3 National Institute for Occupational Safety and Health, Health Effects Laboratory Division, Pathology and Physiology Research Branch, 1095 Willowdale Road, Morgantown, WV 26505, USA; 4 Environmental Protection Agency, E205-09, EPA/ORD, Research Triangle Park, NC 27711, USA; 5 Queen's Medical Research Institute, University of Edinburgh, 47 Little France Crescent, Edinburgh, EH16 4TJ, UK; 6 National Institute of Environmental Health Sciences, MD D2-01, P. O. Box 12233, Research Triangle Park, NC 27709, USA

## Abstract

**Background:**

A symposium on the mechanisms of action of inhaled airborne particulate matter (PM), pathogenic particles and fibers such as silica and asbestos, and nanomaterials, defined as synthetic particles or fibers less than 100 nm in diameter, was held on October 27 and 28, 2005, at the Environmental Protection Agency (EPA) Conference Center in Research Triangle Park, North Carolina. The meeting was the eighth in a series of transatlantic conferences first held in Penarth, Wales, at the Medical Research Council Pneumoconiosis Unit (1979), that have fostered long-standing collaborations between researchers in the fields of mineralogy, cell and molecular biology, pathology, toxicology, and environmental/occupational health.

**Results:**

The goal of this meeting, which was largely supported by a conference grant from the NHLBI, was to assemble a group of clinical and basic research scientists who presented and discussed new data on the mechanistic effects of inhaled particulates on the onset and development of morbidity and mortality in the lung and cardiovascular system. Another outcome of the meeting was the elucidation of a number of host susceptibility factors implicated in adverse health effects associated with inhaled pathogenic particulates.

**Conclusion:**

New models and data presented supported the paradigm that both genetic and environmental (and occupational) factors affect disease outcomes from inhaled particulates as well as cardiopulmonary responses. These future studies are encouraged to allow the design of appropriate strategies for prevention and treatment of particulate-associated morbidity and mortality, especially in susceptible populations.

## Background

Inhaled durable particles and fibers (defined has having a greater than 3:1 length to diameter ratio) have been of concern to human health for over a century due to a myriad of publications reporting lung diseases in trade workers and miners prior to the enforcement of permissible exposure levels in the workplace. Although naturally occurring minerals such as asbestos and silica have been studied clinically and experimentally for many years, it is now clear that inhaled ambient particles also are associated with adverse health effects in man and animals, and their compositional components and biologic effects are now under intense investigation. Because synthetic nanomaterials often occur in size ranges similar to ultrafine airborne particles that are considered to be a major factor contributing to adverse health effects of air pollution, information on the biological reactivity of these particulates is also necessary to allow their safety evaluation.

The goal of this symposium was to understand the mechanisms of action of inhaled particulate matter, known pathogenic minerals, and nanomaterials from presentation of data generated in a number of laboratories in academia, government, and industry. The thematic sessions included: 1) Airborne Particulate Matter-Cardiopulmonary Effects and Host Susceptibility Factors, 2) Metals and Metal Mixtures as Pathogenic Components of Particulate Matter, 3) Mechanisms of Asbestos-Induced Cell Injury and Remediation, 4) Occupational, Environmental and Genetic Factors in the Development of Disease by Fibrogenic Minerals and Cigarette Smoke, and 5) Nanoparticles and Nanomedicine. A recurrent theme throughout the conference was the role of oxidant stress and antioxidant status in response and host susceptibility to particle and fiber effects. Reactive oxygen species (ROS) production is only one of a number of signatures and repercussions of oxidant stress induced by inhaled particles and metals in cells and tissues. Information suggests that biomarkers of oxidative stress and therapeutic strategies targeting ROS and inflammation are ripe areas for prediction of risk and therapy of particle-induced diseases. This synopsis encompasses information presented at platform presentations, although a number of outstanding and relevant poster presentations were also featured at the meeting. We also reference recently published articles by investigators that were covered, in part or full, in their presentations.

This synopsis encompasses information presented at platform presentations, although a number of outstanding and relevant poster presentations were also featured at the meeting. We also reference recently published articles by investigators that were covered, in part or full, in their presentations.

## Results

### Airborne particulate matter – cardiopulmonary effects and host susceptibility factors

Airborne particulate matter (PM) from vehicle exhaust, combustion, road dust and windblown soil, has been associated with increases in respiratory and cardiac morbidity and mortality. PM can occur as coarse particles with a diameter of more than 2.5 μm that generally are derived from natural sources such as soil and sea salts, whereas fine (0.1 to 2.5 μm in diameter) and ultrafine (< .1 μm in diameter) PM occurs as by-products of combustion of fossil fuels. The complexity and seasonal variations of the many components of PM which include metals, nitrates, sulfates, and organic hydrocarbons, make it difficult to identify the precise toxic species that contribute to inflammation, lung disease and cardiovascular effects. However, ultrafine particles, a major component of vehicle emissions that are increased in urban air, may be more pathogenic after inhalation as they have a large surface area per unit mass, a proportionally higher content of toxic hydrocarbons, and increased ability to penetrate into lung tissue. Moreover, inhalation of PM interferes with bacterial inactivation and clearance from the lung.

Clinical ramifications of high PM exposures include airway inflammation, exacerbation of asthma, chronic bronchitis and airway obstruction. Large cohort studies also suggest that pollution may increase lung cancer risk [1]. In addition, cardiovascular effects of episodic high levels of PM include stroke, heart attacks, heart arrhythmias, and sudden death. Healthy volunteers as well as potentially susceptible populations, including the elderly and individuals with asthma or chronic obstructive pulmonary disease (COPD), have been evaluated to understand the mechanisms of cardiopulmonary dysfunction. Exposure to PM from brake wear and engine emissions in cars and patrol vehicles is associated with cardiovascular effects in healthy young men [2] and onset of myocardial infarction [3], and age and other susceptibility factors may exacerbate risks.

Both clinical and animal studies implicate oxidative stress and impairment of antioxidant defenses as risk factors in PM-induced cardiovascular responses. Recently, examination of subjects for changes in heart rate variability (HRV) revealed that effects of PM occurred predominantly in people who were missing the glutathione S-transferase M1 (GSTM1) gene, or were obese or hypertensive [4]. Based upon interactions between the development of obesity and asthma, obesity might also be a risk factor in PM-induced exacerbation of asthma. Diabetes also enhances PM-associated impairment in vascular reactivity and endothelial function [5]. Increases in PM did not affect HRV in patients on angiotensin converting enzyme (ACE) inhibitors or statins, suggesting that these treatments might ameliorate the adverse cardiac effects of PM.

Although several groups of researchers have shown that cardiac effects can occur in rodents and dogs after acute exposures to concentrated airborne particulates (CAPS), a recent study demonstrates that long-term (6 month) exposure to New York PM at approximately 10× ambient concentrations enhances the formation of atherosclerotic plaques and altered vasomotor tone in susceptible, atherosclerosis-prone mice (Apo E-/- mice) [6]. The fact that atherosclerosis was accelerated in these mice also demonstrating increases in lung and vascular inflammation, suggests that lung-generated cytokines or other mediators may systemically activate inflammatory cascades in vessel walls. These signaling and inflammatory pathways need to be characterized both in cells of the lung and in the cardiovascular system.

How airborne particulates initially deposited in the lung might influence the formation of thrombi in the peripheral circulation has been explored in a hamster model of femoral vein thrombus formation [7]. In this model, intratracheal administration of diesel exhaust particles (DEP) leads to enhanced thrombus formation within one hour, a phenomenon attributed to translocation of particles from the lungs into the circulation. The prothrombotic effects of DEP were associated with platelet activation and mitigated by pretreatment with diphenhydramine (an H1-histamine receptor antagonist), dexamethasone or sodium cromoglycate, a mast cell stabilizer.

Transition metal content of PM has emerged as an important risk factor in the prevalence of allergic asthma in children and airway inflammation/oxidative stress in lungs of healthy subjects. Toxicogenomic and proteomic studies [8-10] in animal and cell culture models of PM exposure show that both metal ion content and oxidation state are important in determining the bioreactivity of PM. After intratracheal instillation into rats of metal ions identified in the soluble fractions of both historic and present day London PM samples [8], gene profiling of lung and heart tissues demonstrates transient changes in gene expression, primarily associated with xenobiotic metabolism and antioxidant recycling, in heart that are less robust than changes found in the lung.

An *in vitro *cellular hierarchical oxidative stress model using proteomics demonstrates 3 tiers of dose-related responses to DEP, organic DEP extracts, and ambient ultrafine particles [11]. At the lowest levels of oxidative stress (Tier 1), induction of phase II antioxidant and detoxification enzymes occurs that involves activation of the transcription factor Nrf2, which drives the antioxidant response element (ARE) in the promoter of phase II genes. These phase II enzymes protect against PM-induced proinflammatory (Tier 2) and cytotoxic (Tier 3) effects at higher concentrations. However, compromised Tier 1 responses or exacerbated Tier 2 and 3 responses may increase individual susceptibility to DEP whereas enhanced phase II enzyme expression may promote adaptation or resistance.

The net outcome of these responses in lung may govern the extent of small airway remodeling or disease that occurs after high level chronic exposures to PM. These mechanistic events are being elucidated in a rat tracheal organ culture model after exposure to PM or DEP [12]. In this model, both PM and DEP induce procollagen gene expression and increased hydroxyproline, a marker of collagen deposition that can be prevented with the TGF-β1 antagonist, fetuin (α2-HS-glycoprotein). Experiments using both particulates and cigarette smoke indicate that oxidative activation of TGF-β1 is a critical feature of small airway remodeling by all agents.

Novel *in vivo *imaging approaches demonstrate oxidative stress directly in heart and in the spinotrapezius muscle of rats after intratracheal instillation of residual oil fly ash (ROFA). Oxidative stress in the systemic microvascular wall coincides with adhesion of PMNs, deposition of myeloperoxidase (MPO), and impairment of endothelium-dependent arteriolar dilation [13,14]. These results are consistent with studies showing increases in lung and heart chemiluminescence, an indication of oxidant stress, and tissue-specific increases in activity of antioxidant enzymes [15], suggesting adaptive or defense responses in rats after inhalation of Concentrated Airborne Particulates (CAPS). The fact that administration of N-acetylcysteine prevents oxidative stress measured by accumulation of thiobarbituric reactive substances in lung, as well as lung inflammation in this model [16], implicates oxidants as mediators of acute effects of PM in lung.

### Metals and metal mixtures as pathogenic components of PM

The major metallic components of PM include iron, silicon, aluminum, copper, and zinc, and a number of studies suggest that some of these metals, especially iron, initiate redox reactions that are implicated in inflammation and adverse health effects. PMs and their metal components can be regionally very different, and not all PM from individual regions contains the same amount and type of metal. Thus, the precise delineation of certain metals as mediators of inflammation and disease causation is difficult. Moreover, interactions between metal mixtures and other components of PM may also be confounding. Signaling pathways elicited by metallic components of PM, including zinc (Zn^2+^) and vanadium (V^5+^), also may be critical in redox regulation of protein tyrosine phosphatases (PTPs) inactivating the mitogen activated protein kinases (MAPKs) or epidermal growth factor receptor (EGFR) [17,18]. In human airway epithelial cells *in vitro*, these metals stimulate kinase activity by impairing EGFR and MAPK-directed dephosphorylation, probably by a redox mechanism attacking sulfhydryl (SH) groups on phosphatases. Thus, Zn^2+ ^and V^5+ ^appear to inhibit critical phosphatases that normally control aberrant signaling events leading to cell proliferation and stress rather than acting directly to stimulate kinase phosphorylation. These findings may be important in the design of preventive and therapeutic strategies to inhibit signaling pathways initiating altered epithelial cell function in lung disease.

Vanadium compounds also occur in ROFA and may be linked to the development of occupational asthma and bronchitis. After inhalation of vanadium pentoxide (V_2_O_5_), airway remodeling including mucous cell metaplasia and fibrosis, are observed in rodents [19]. Gene profiling of lung fibroblasts exposed to V_2_O_5 _*in vitro *reveals immediate increases in NADPH oxidase mRNA levels accompanied by release of H_2_O_2_. These events precede early gene responses (1 to 4 hours) that include increased mRNAs for number of growth factors (heparin-binding epidermal growth factor or HB-EGF, etc.) and later gene responses (after 12 hours) reflecting increased expression of vascular endothelial growth factor (VEGF), interleukins and receptors (IL-1R) and complement C9. Transgenic models using COX-2 [20] or STAT-1 [21] null mice [21] show that these mice are protected from V_2_O_5 _-induced fibrosis. *In vitro *work suggests that epithelial production of soluble mediators promotes proliferation and collagen production by smooth muscle cells and fibroblasts in this model of fibrogenesis.

Genetic linkage analysis of nickel-induced acute lung injury in mice has identified as many as five interacting loci that may be related to distinct pathophysiologic alterations in this model. One transcriptional change is a large increase in metallothionein (Mt) gene expression, a gene in proximity to one of these linkage regions. Gene profiling also reveals that genes involved in TGF-β1 signaling and increases in TGF-β1 in bronchoalveolar lavage fluid (BALF) are increased in mice inhaling nickel sulfate whereas down-regulated genes include those involved in lung fluid adsorption or surfactant and lipid synthesis [22]. Mt 1/2 null mice are more susceptible to nickel-induced pulmonary inflammation, injury, and lethality than Mt 1+/2+ mice, whereas Mt-overexpressing transgenic mice have an increased survival rate [23]. Gene expression associated with inflammation and changes in extracellular matrix or coagulation/fibrinolysis is greater in nickel-exposed Mt1/2 null mice compared to Mt 1+/2+ mice. These studies establish metallothionein as a protein critical to host defense in acute lung injury by nickel.

Iron is an integral component of pathogenic amphibole (crocidolite, amosite) asbestos fibers, occurs in samples of PM, and is often a contaminant of silica dusts in South African gold mines. Because iron drives a modified Haber-Weiss or Fenton reaction leading to generation of hydroxyl radical (^. ^OH), it may contribute to the biological reactivity of particle mixtures. For example, when aerosols of soot and iron particles in the ultrafine range are inhaled by healthy adult rats, animals exposed to soot or iron alone for 3 days have no adverse respiratory effects. However, addition of approximately the same concentrations of iron to soot in combined exposures causes synergistic increases in pulmonary oxidative stress, elevations in IL-1B and activation of the transcription factor nuclear factor-κB (NF-κB), an oxidant-induced transcription factor [24]. These studies suggest that metals and particle mixtures may have increased reactivity that may be more than the sum of individual agents alone.

Metals such as chromium occur in mixtures of foundry dusts or in PM and may sensitize airway epithelium to injury from secondary insults such as arsenic or inflammatory cytokines. *In vitro *studies shows that CrVI suppresses activation of a number of transcription factors (NF-κB, AP-1, Nrf2) in human airway epithelial cells that led to loss of inducibility of protective genes encoding proteins such as heme oxygenase-1 [25]. Although CrVI suppresses many genes, it does not globally suppress constitutive or inducible gene expression. However, CrVI stimulates phosphorylation of STAT family proteins and causes chronic induction of STAT-driven genes.

The silencing of protective genes required for defense from secondary inflammatory insults by CrVI at nontoxic concentrations may be a subtle effect occurring at low levels of CrVI-containing particulates.

### Mechanisms of asbestos-induced cell injury and remediation strategies

Asbestos fibers are causally linked to the development of lung cancers, malignant mesotheliomas and pleural and pulmonary fibrosis (asbestosis) and have been incorporated into thousands of industrial products worldwide. Although asbestos has been banned in several Western European countries, it continues to be used in developing countries and persists in buildings and commerce. Tons of naturally occurring asbestos fibers also exist in mineral ores and soils, thus prevention of future asbestos-associated disease will depend largely upon new strategies for remediation and detoxification of "in place" asbestos. We have known for several years that the active iron ions on amphibole types of asbestos, such as crocidolite or amosite, cause sustained generation of reactive oxygen species (ROS) that are related directly to fiber toxicity and effects on DNA and proteins [26]. Moreover, some samples of chrysotile asbestos contain small amounts of iron that substitute for magnesium ions in its structure. Detoxification and removal of naturally occurring asbestos in an outdoor environment or asbestos products in buildings may require unique strategies. Several new approaches requiring collaboration between chemists and biologists include spontaneous polymerizations of natural products on the surface of asbestos fibers and bioremediation by soil fungi or lichens that adhere to fibers and selectively remove iron [27]. These amorphous asbestos fibers may prove to be less durable and non-pathogenic, requiring validation in *in vitro *and animal testing.

Asbestos-associated diseases continue to be devastating worldwide, and new biomarkers for early detection of asbestos-induced cancers and fibrosis are sorely needed. We continue to make progress on understanding the critical events in asbestos-induced carcinogenesis and fibrogenesis with hopes of applying these findings to the development of biomarkers and improved therapies for individuals with lung cancer, mesothelioma and fibrosis. Asbestos is a carcinogen that both damages DNA and causes changes in the proliferation and differentiation of target cells of lung and pleural diseases. Chromosomal deletions and loss of tumor suppressor genes have been reported in many mesotheliomas, and loss of Betaig (Big)-h3 protein is frequent in primary lung carcinomas [28]. In an immortalized human bronchial epithelial cell line, chrysotile asbestos induced neoplastic transformation and growth of cells in nude mice. Microarray studies revealed that the *BigH3 *gene is consistently silenced in tumor cells due to methylation of its promoter region. The demonstration that re-expression of *BigH3 *suppresses tumor growth suggests that this tumor suppressor gene plays a critical role in fiber-induced carcinogenesis.

p53 is another tumor suppressor gene implicated in the development of asbestos-induced mesotheliomas and linked more recently to asbestos-induced mitochondrial damage and apoptosis of alveolar epithelial cells [29]. Exciting results showed that amosite asbestos activates p53 promoter activity and increased levels of p53 mRNA and protein. Moreover, asbestos-induced p53 expression in epithelial cells at the broncho-alveolar duct junctions of asbestos-instilled rat lungs was blocked by phytic acid, an iron chelator that may act directly on fibers or cells to block redox reactions. The fact that p53-dependent transcription regulates asbestos-induced apoptosis through a mitochondrial death pathway that may be abrogated *in vivo *by an iron chelator may have broad implications in abrogating epithelial cell injury critical to the development of fibrogenesis or carcinogenesis.

Several signaling pathways including NF-κB, Protein kinase C, and the mitogen activated protein kinases (MAPK) are activated in lung epithelial cells *in vitro *or after inhalation of asbestos fibers. The extracellular signal regulated kinases (ERK1/2) have been intensely studied because this MAPK pathway, as opposed to the c-Jun-N-terminal kinases (JNKs) or p38 cascades, is selectively phosphorylated in lung epithelium by asbestos through a redox-active process. The duration of ERK1/2 signaling governs survival and death responses to both asbestos and chemical generating systems of ROS and reactive nitrogen species (RNS), presumably through events promoting expression of cyclin D1 and cell cycle progression at low concentrations, and stabilization of c-Fos in the cell nucleus, preventing expression of cyclin D1 and promoting epithelial apoptosis through apoptosis-inducing factor at higher, toxic concentrations [30]. Increased cyclin D1 and Ki-67expression, an indication of cell cycle progression, are observed in mice after inhalation of chrysotile asbestos, and these markers of proliferation are increased in asbestos-exposed MPO null mice in comparison to MPO sufficient, wild type mice that exhibited more lung inflammation [31]. These results suggest that MPO as an oxidant generating enzyme primarily found in neutrophils that may regulate epithelial responses to asbestos.

The ERK1/2 pathway has recently been linked to downstream effects of Tumor necrosis factor-α(TNF α), a cytokine critical to TGF-β1 expression [32]. TNFα receptor null mice are resistant to chrysotile asbestos-induced fibrosis, but overexpression of latent TGF-β1 prior to inhalation of asbestos by TNFα receptor null-resistant mice causes the development of lesions of the same severity observed in asbestos-exposed wild type mice. These effects are attributed to oxidant-associated activation of the latent TGF-β1 peptide suggesting a mechanism through which ROS-generating particulates induce fibrogenesis and lung remodeling. Global and lung epithelial cell-specific targeting of ERK1/2 activation by pharmacologic strategies and protein targeting might reveal whether asbestos-induced epithelial cell responses and lung remodeling are modified in murine models of asbestosis.

### Occupational, environmental and genetic factors in the development of pulmonary disease by fibrogenic minerals and cigarette smoke

Like asbestos, respirable quartz particles and silica-containing coal dusts may damage DNA at high concentrations by eliciting DNA strand breakage and formation of the oxidative DNA adduct, 8-hydroxydeoxyguanosine (8OHdG), in lung epithelial cells *in vitro *that can be abrogated by surface coatings of quartz particles with polyvinylpyrimidine-N-oxide or aluminum lactate. *In vivo *ROS from inflammatory cells may also mediate DNA damage or apoptosis in lung epithelium followed by enhanced expression of the base excision repair protein, apurinic apyridiminic endonuclease (APE/Ref-1) [33]. Since APE-1/Ref-1 has recently been functionally characterized as a DNA repair enzyme in spontaneous DNA damage in human cells [34], individual susceptibility to silica and oxidant generating particulates may reflect the efficiency of DNA repair by this and other pathways.

Inhaled silica and other particles such as residual oil fly ash (ROFA) or stainless steel welding fumes (SSWF) that generate ROS [35] may alter lung defense to bacterial and other infections. In a rat bacterial infectivity model of *Listeria monocytogenes*, pre-instillation of ROFA or SSWF slowed lung clearance of bacteria and increased animal morbidity, effects attributed to soluble and insoluble metal components [36]. In contrast, silica-enhanced lung bacterial clearance strikingly upregulated oxidant production and activated neutrophils and alveolar macrophages (AM) [37]. These results suggest that pro-oxidant effects of metal components of pathogenic dusts may lead to altered lung defense mechanisms in combating infections.

The AM is a critical cell type that is associated with lung defense but also produces ROS in response to silica and asbestos. Human and murine AM also undergo apoptosis in response to silica, and silica-induced responses and fibrosis in mice can be prevented by modulation of scavenger receptor A (CD204) and MARCO (another member of the Class A scavenger receptors). Silica-induced apoptosis and ROS production that may be a consequence rather than a cause of apoptosis are ablated in AM from CD204/MARCO double null mice which continue to phagocytose silica particles [38]. Another signaling pathway critical to cytoskeleton-mediated quartz particle uptake in AMs is one involving the FcγII receptor involving recruitment of small GTPases in an actin-mediated process [presented by P. Haberzettl, University of Dusseldorf, Dusseldorf, Germany]. Use of cytochalasin B inhibits uptake of quartz, ROS generation, and release of TNFα, suggesting a critical link between particle uptake and production of inflammatory mediators. Understanding how various receptors (alone and together) signal responses of AM to fibrogenic particles, possible cross-talk between these signaling pathways, and whether the distribution of scavenger and other receptors differs in functionally different subsets of AM will be critical to deciphering pro-inflammatory from lung defense responses to inhaled particulates.

Cigarette smoke, a complex mixture of chemicals and particulates, is a co-factor in the development of asbestos and silica-induced pulmonary fibrosis and lung cancers, and rodent and human bronchial epithelial cells (1HAEo and HBE1) *in vitro *exhibit alterations in differentiation including induction of SPRR1B, a marker of squamous metaplasia that is an early lesion in the pathway to carcinoma. Stimulation of the c-Jun and Fra-1 based activator-protein-1 (AP-1) transcription factor is a critical event leading to squamous metaplasia in bronchial epithelial cells. In a freshly generated mainstream smoke exposure model, Fra-1 expression was regulated by a metalloproteinase (MMP) and epidermal growth factor receptor (EGFR)-dependent MAPK pathway. Another outcome of this cascade is activation of a phosphoinositol-3-kinase (PI3K), AKT-independent mechanism that appears to regulate extracellular signal regulated kinase (ERK1/2) MAPK activation and subsequent Fra-1 [39]. In contrast, the transcription factors, Elk-1 and cAMP response element binding protein (CREB), appear to be more important in cigarette smoke-regulated Fra-1 and AP-1 transactivation. These studies show that multiple transcription factors may cooperate in responses of bronchial epithelium to inhaled toxic particles.

Both genetic and occupational/environmental factors such as cigarette smoking and exposure to particulates are critical to the development of pulmonary fibrosis. Combined clinical studies and genomic profiling are likely to reveal interactions between specific environmental agents and genes predisposing to idiopathic pulmonary fibrosis (IPF) and subtypes of idiopathic interstitial pneumonia (IIP) [40]. In a recent study where families with two or more cases of IIP occurred in first degree family members, 111 families with familial interstitial pneumonia (FIP) having 301 affected and 360 unaffected individuals were evaluated. After controlling for age and gender, smoking was strongly associated with the development of FIP, and 20 pedigrees demonstrated vertical transmission consistent with autosomal dominant inheritance, with some pedigrees demonstrating several subtypes of IIP occurring within the same families. These exciting results suggest that gene-environmental interactions are critical to host susceptibility in FIP and pulmonary fibrosis.

### Nanoparticles and nanomedicine: a new and challenging frontier

Nanotechnology is the manipulation of matter on a near-atomic size scale (~ 1–100 nm) to engineer new structures, materials, and devices. This technology will impact numerous strategies in the field of cardiopulmonary medicine. Research in nanoscale materials is growing rapidly worldwide, and by 2015, nanotechnology is expected to have a one trillion dollar impact on the global economy. Considering the rapid growth of nanotechnology and the variety of nanomaterials potentially used in the future, identifying, quantifying and managing potential health risks is essential, especially in the respiratory tract that may serve as the portal of entry for inhaled nanoparticles and nanofibers.

Many published studies on PM or particles comprising PM support the generalization that ultrafine particles are more cytotoxic, inflammatory and fibrogenic on an equivalent mass basis than fine-sized particles of the same composition. While the bioreactivity of ultrafine particles is largely accepted as a probable indication of disease potential, the toxic potential of a nanoparticle is also affected by surface treatments/coatings, degree of agglomeration, particle shape, and/or surface charge. It is likely that hazards of newly designed nanoparticles will need to be evaluated on a case-by-case basis once screening strategies are in place [41].

Carbon nanotubes are a unique class of synthetic structures being evaluated for improved electrical, mechanical, and thermal properties in the aerospace, electronic and computer industries. After pharyngeal aspiration of single-walled carbon nanotubes, a technique that introduces a large bolus of fibers in contrast to more physiologic inhalation routes, acute oxidant stress, inflammation, and granulomas developing at deposition sites of nanotube aggregates are observed [42]. Early-onset proximal fibrosis and depletion of glutathione in bronchoalveolar lavage fluid (BALF) occurs within seven days, and TGF-β1 levels in BALF peak at seven days. Diffuse interstitial fibrosis progressed from seven to 60 days in the distal lung, a phenomenon attributed to translocation of nanotubes to distal alveolar septa. Experiments using aspiration of equal mass amounts of ultrafine carbon black or silica revealed less acute epithelial injury and neutrophil recruitment than that observed with carbon nanotubes, suggesting different kinetics of lung injury. Although particle delivery in these studies appears more diffuse than intratracheal instillation studies, inhalation studies using lower dose ranges of nanomaterials over time are advocated for more precise evaluation of toxicity in relevant airborne exposures.

Although it is clear that the lung will be an initial target of inhaled nanomaterials, their small size enables their translocation via pulmonary capillaries and other mechanisms to systemic sites. Thus, revealing the systemic effects of nanoparticles in the vasculature, distal organs and sensory nervous system, since it has been shown that ultrafine particles can be transported to the olfactory bulb in the brain via sensory nerves in the nasal cavity [43], is critical to understand the systemic effects of nanoparticles in the vasculature, distal organs and sensory nervous system. The derivation of pulmonary and cardiovascular effects of combustion-derived nanoparticles (CDNP), such as diesel exhaust particles and welding fumes [44], appear to follow paradigms previously documented with asbestos and silica as well as metal-containing PM, namely that oxidative stress and inflammation are critical to the initiation of adverse pulmonary reactions. The contribution and identification of inflammatory mediators generated in the lung or BALF and/or after translocation of ultrafine or nanoparticles from the lung to blood, lymphatics and other organs need definition. Another critical target may be endothelial cells as inhalation of diesel exhaust particles alters endothelial function in the systemic circulation. The origin of changes in autonomic innervation, heart rate variability, and electrical activity of the brain, measured by quantitative EEG approaches in healthy human volunteers inhaling diesel exhaust, may also involve a combination of systemic effects induced by inhaled and translocated ultrafine particles [B. Crut et al., Zuyd University, Heerlen, The Netherlands, manuscript in preparation].

The large internal and external surface area of synthetic nanoparticles makes them useful tools for drug delivery to the lung and heart. In addition, labeled or magnetic nanoparticles can be employed in high resolution imaging to increase local retention and proliferation of fibroblasts after intra-arterial injections targeting ischemia [presented by PJA Borm, Zuyd University, Heerlen, The Netherlands].

Although the extremely small size of nanomaterials permits their entry into the circulation and transport potentially to other organs where uptake may also occur, synthetic particles can be engineered in larger size ranges with defined pore dimensions for optimum drug delivery and direct targeting to lung or pleura. Thus nebulization or intrapleural injection circumvents systemic drug toxicity, a central problem in patients with lung cancers and mesotheliomas. Acid-prepared mesoporous spheres (APMS) (1–2 micron diameter with 40 A diameter pores) have been examined in preclinical studies and are nontoxic and noninflammatory after intrapleural injection or intranasal administration into mice [45]. APMS increases both the delivery and killing potency of doxorubicin (DOX) in human mesothelioma cells and are phagocytized by an actin-dependent process evading phagolysosomes. Tumor prevention studies in a nude mouse xenograft model using human mesotheliomas are currently in progress. These approaches and studies summarized above using nanomaterials are strengthened by collaborations between chemists, biologists, toxicologists, and clinicians.

## Conclusion

Occupational exposures to inhaled pathogenic particles and fibers such as silica and asbestos have been associated with the development of lung inflammation and disease. The development of lung fibrosis and cancer by these naturally occurring minerals is dose and time-related with relatively long durations of exposure. In the last decade, epidemiology and animal models have revealed acute morbidity and mortality, including exacerbation of asthma and toxic effects extending to the cardiovascular system, from episodic exposures to PM. Dissecting the adverse components of PM and mechanistic events occurring in the lung, its initial site of deposition, and systemically is daunting because of the complexity and seasonable variability of PM. However, ultrafine particles and components such as metals (iron, vanadium, zinc, etc.), sulfates, and adsorbed hydrocarbons may act uniquely or synergistically and/or render the lung more sensitive to secondary insults. The majority of experimental data suggests that size and pro-oxidation state of PM determine its overall distribution and durability in lung and distal organs, cell/tissue transport mechanisms and toxic potential. This information may be vital to the design and use of "safe" synthetic nanoparticles and nanofibers, a burgeoning technology.

Proinflammatory and acute effects of PM are directly related to oxidative stress and induction of multiple oxidant or xenobiotic-induced signaling cascades and transcription factors (NF-κB, AP-1, Nrf2), a paradigm consistent with models of asbestos and silica-associated lung diseases. An important consequence of initial oxidant injury is the antioxidant response that when overwhelmed or compromised in subjects with weakened defenses, i.e., individuals with single nucleotide polymorphisms or changes in expression of antioxidant enzymes, the GSTM1 gene, etc., predisposes to cardiopulmonary morbidity or mortality. Information on other host susceptibility factors and genes predisposing to the development of acute and chronic diseases by inhaled particles may be obtained by genotype and phenotype analyses.

Knowledge of the events leading to systemic transport and pathogenicity of PM, including impairment of vascular and fibrinolytic function, is also vital to preventive approaches in the public health domain. The presentations summarized above suggest a number of potential mechanisms whereby PM may adversely affect the cardiovascular system by altering autonomic function, vascular reactivity, and thrombosis. However, how these effects contribute to and whether they are related causally to the pathogenesis of atherosclerosis are unclear. Another important question that needs to be addressed is whether the physical presence of PM is required for distal organ effects or whether these effects are mediated by lung chemokines, cytokines or inflammatory cells after inhalation of PMs. Moreover, the many physical and chemical properties of PM and other inhaled particulates that are central to their pathogenicity need to be elucidated.

## Abbreviations

ACE Angiotensin converting enzyme

AM Alveolar macrophages

AP-1 Activator-protein-1

APE/Ref-1 Apurinic apyridiminic endonuclease

APMS Acid-prepared mesoporous spheres

Apo E-/- mice Atherosclerosis-prone mice

ARE Antioxidant response element

BALF Bronchoalveolar lavage fluid

CAPS Concentrated airborne particulates

CDNP Combustion-derived nanoparticles

COPD Chronic obstructive pulmonary disease

CREB cAMP response element binding protein

DEP Diesel exhaust particles

EGFR Epidermal growth factor receptor

ERK1/2 Extracellular signal regulated kinases

FIP Familial interstitial pneumonia

GSTM1 Glutathione S-transferase M1

HB-EGF Heparin-binding epidermal growth factor

HRV Heart rate variability

8OHdG 8-hydroxydeoxyguanosine

IIP Idiopathic interstitial pneumonia

IL-1R Interleukin-1 receptor

IPF Idiopathic pulmonary fibrosis

JNKs c-Jun-N-terminal kinases

MAPKs Mitogen activated protein kinases

MMP Metalloproteinase

MPO Myeloperoxidase

Mt Metallothionein

NF-κB Nuclear factor-κB

PI3K Phosphoinositol-3-kinase

PM Particulate matter

PTPs Protein tyrosine phosphatases

RNS Reactive nitrogen species

ROFA Residual oil fly ash

ROS Reactive oxygen species

SSWF Stainless steel welding fumes

TNFα Tumor necrosis factor-α

VEGF Vascular endothelial growth factor

## Competing interests

No authors have competing interests. The findings and conclusions of this report have not been formally disseminated by NIOSH, NIEHS or the EPA and should not be construed to represent any agency determination or policy.

## Authors' contributions

All authors participated in the design and coordination of this meeting and helped to draft the manuscript. All authors read and approved the final manuscript.
